# Co-developing a tailored vaccination intervention with Congolese migrants: a participatory study

**DOI:** 10.1093/eurpub/ckac131.107

**Published:** 2022-10-25

**Authors:** AF Crawshaw, C Hickey, LM Lutumba, LM Kitoko, SL Nkembi, F Knights, Y Ciftci, T Vandrevala, AS Forster, S Hargreaves

**Affiliations:** 1 Institute for Infection and Immunity, St George’s, University of London, London, UK; 2 Hackney Refugee and Migrant Forum, Hackney CVS, London, UK; 3 Hackney Congolese Women Support Group, London, UK; 4 Doctors of the World UK, London, UK; 5 Faculty of Health, Social Care and Education, Kingston and St George’s, University of London, London, UK; 6 Our Future Health, Manchester, UK

## Abstract

**Background:**

Disparities in vaccination uptake among migrant populations are well documented. WHO and ECDC have sought renewed focus on participatory research that engages migrants in co-producing tailored initiatives to address vaccination inequities and increase coverage.

**Methods:**

This community-based participatory research study aims to engage Congolese migrants in co-developing a tailored approach to increase vaccine uptake. Phase 1 used poster walls and in-depth interviews with Congolese migrants (n = 32) to explore COVID-19 vaccination beliefs, experiences, and preferred information sources and communication methods, analysed iteratively and thematically in NVivo.

**Preliminary results:**

Institutional distrust has shaped this population’s interpretation of the pandemic response and enabled vaccine misinformation and conspiracy theories to take hold. We found complex information networks and preference for Francophone, African and social media. Limited English proficiency and preference for the oral tradition restricted engagement with official public health messaging. Suspicion of government motives, low knowledge, and culturally specific perceptions about vaccination contributed to belief that breakthrough infections and need for COVID-19 boosters imply the vaccine is not effective. The population felt coerced by vaccination reminders and mandates, and were resultantly more hesitant to accept COVID-19 vaccination.

**Conclusions:**

The population’s specific characteristics suggest that existing and trusted interpersonal networks and oral communication in first languages should be harnessed to spread credible information and encourage vaccine uptake, and mandate policies are unlikely to be effective. Training local role models to facilitate vaccination dialogues and myth-bust may be effective at changing behaviour. The next phases will gather more information from key stakeholders and engage migrants in workshops to co-design insight-driven, tailored interventions.

**Key messages:**

• Global policy-setting organisations have called urgently for participatory research that engages migrants in the co-production of tailored initiatives to address vaccination inequalities.

• Populations with strong interpersonal networks and low trust in public institutions may be receptive to tailored, community-centred dialogue approaches using local messengers and role models.

